# BLOOD-BASED BIOMARKER CHANGES IN A PHASE 2B TRIAL ASSESSING LOMECEL-B IN OLDER ADULTS WITH FRAILTY

**DOI:** 10.1093/geroni/igac059.2121

**Published:** 2022-12-20

**Authors:** Kevin Ramdas, Danial Mehranfard, Eric Naioti, Geoff Green, Lisa McClain-Moss, Dan Gincel, Joshua Hare, Anthony Oliva

**Affiliations:** Longeveron Inc., Miami, Florida, United States; Longeveron Inc., Miami, Florida, United States; Longeveron, Inc., Miami, Florida, United States; Longeveron Inc., Miami, Florida, United States; Longeveron Inc., Miami, Florida, United States; Longeveron Inc., Miami, Florida, United States; Longeveron Inc., Miami, Florida, United States; Longeveron Inc., Miami, Florida, United States

## Abstract

Aging frailty (AF) is a multidimensional geriatric syndrome that is characterized by physical and cognitive symptoms, increasing the vulnerability of affected older adults to adverse health outcomes. Mechanistically, a low-grade chronic inflammatory state (inflammaging), endothelial dysfunction, and decreased regenerative capacity are thought to be major contributors to AF pathophysiology. Lomecel-B is an allogenic medicinal signaling cell(MSC) formulation that can potentially ameliorate AF through pleiotropic mechanisms, including anti-inflammatory, pro-vascular, and pro-regenerative activities. We completed a Phase 2b randomized, double-blinded, placebo-controlled trial designed to assess Lomecel-B benefits for AF via change versus placebo in the six-minute walk test(6MWT), to assess the dose-response relationship, and to evaluate bioactivity via changes in blood-based biomarkers. Enrolled subjects were aged 70–85 years with mild-to-moderate AF, a reduced 6MWT of 200-400m, and Tumor Necrosis Factor-α of ≥ 2.5pg/mL indicative of inflammaging. In total, 143 subjects received a single intravenous infusion of Lomecel-B at doses of 2.5 x 107 cells (25M, N=35), 5.0 x 107 cells (50M, N=30), 1.0 x 108 cells (100M, N=33), or 2.0 x 108 cells (200M, N=16), or placebo (N=29). Safety and efficacy assessments were performed at 1, 3, 6, and 9 months post-infusion. Increases in 6MWT and decreases in serum levels of the blood-based biomarker Soluble-Tie-2 were observed at 9 months in the Lomecel-B groups versus placebo. Notably, both observations were seen in a dose dependent fashion with 200M showing the highest effect. Based on the findings, a next-phase trial is being developed to advance this clinical program and will be presented.

